# Predicting family doctor contract fulfillment propensity using the FA-GA-BP model and per capita household expenditures

**DOI:** 10.3389/fpubh.2025.1709701

**Published:** 2026-01-08

**Authors:** Qiaowen Tang, Daisheng Tang

**Affiliations:** School of Economics and Management, Beijing Jiaotong University, Beijing, China

**Keywords:** family doctor contract services, contract fulfillment propensity, per capita living expenditure, factor analysis, neural network

## Abstract

**Background:**

This study aimed to identify the propensity to fulfill family doctor contract services (FDCS) among community residents and to develop a low-error, high-precision inversion model. The development of this model is crucial for monitoring the quality of FDCS and advancing basic community health services.

**Materials and methods:**

Based on a survey of a typical urban community, this study used average per capita household living expenditure as the primary input parameter. Data on FDCS fulfillment frequency from six consecutive quarters across communities were analyzed. The study combined factor analysis (FA) with genetic algorithm (GA) optimization of the backpropagation (BP) neural networks to simulate fulfillment tendencies for FDCS. The model's accuracy and applicability were then evaluated. FA of per capita household living expenditure identified two principal factors significantly influencing FDCS fulfillment propensity: “Quality of Life Factor” and the “Rigid Demand Factor.” Extracting these factors from per capita expenditure via FA and then employing the BP algorithm for simulation significantly improved estimation accuracy relative to conventional BP models using unsimplified parameters.

**Results:**

The prediction values obtained from the combined FA and GA–BP method yielded a coefficient of determination $R_{GA\text{\_}BP}^{\text{2}}=0.9223$ with the measured values. The root mean square error and RE relative error of the fitted model were 0.0618 and 9.20%, respectively. Communities were classified by fulfillment rates and subjected to FA–GA–BP simulation predictions to meet management needs. The simulated coefficient of determination for all classifications exceeded 0.8138.

**Conclusion:**

The findings indicate that the FA–GA–BP model provides reliable and generalizable predictions of the propensity to fulfill FDCS among residents. This robust inversion model, developed by optimizing the BP algorithm with FA and GA, exhibits high accuracy, low error, and good compatibility with data from diverse community datasets. The results have significant implications for the dynamic monitoring of FDCS fulfillment status.

## 1 Introduction

The family doctor system serves as the core model for primary healthcare globally. The General Practitioner system in the United Kingdom provides universal coverage for initial consultations and health management services (1). The United States Accountable Care Organizations use performance-based incentives to enhance service quality (2). The World Health Organization (WHO) advocates for Primary Health Care grounded in family doctors (3). In China, primary care began in 2009, and comprehensive family doctor contract services (FDCS) were launched nationwide in 2016. By 2023, the contract rate exceeded 40%, with coverage for high-priority groups (older adult, patients with chronic disease, etc.) surpassing 70%. Policy initiatives have promoted the “1+1+1” contract model (1 community hospital + 1 district-level hospital +1 municipal-level hospital). Nonetheless, challenges remain, including service homogenization and insufficient public awareness (4–6). In practice, “contracts without fulfillment” have hindered policy implementation (7, 8). The fulfillment propensity of FDCS refers to the willingness and behavioral tendencies of contracted residents to continuously receive and actively participate in health management services provided by family doctors. This indicator is key to assessing the effectiveness of contracted services, affecting both the quality and efficiency of primary healthcare delivery. Household consumption levels directly influence health expenditures (9). Numerous studies indicate that households with higher economic status tend to increase their healthcare spending, often through regular physical examinations, commercial insurance, or premium medical services; by contrast, households with lower income status may delay medical care or rely on basic medical insurance because of budget constraints (10, 11). Evidence suggests that a 10% rise in consumption expenditure correlates with a 3%–5% increase in healthcare spending [(12), WHO Health Equity Report/China Household Finance Survey]. Since the implementation of family doctor policies, promoting service enrollment has remained a key priority. However, effective evaluation of contract fulfillment is still lacking. The propensity for patients to fulfill their contracts is a critical factor for FDCS sustainability. Owing to socioeconomic disparities among residents in different communities, the specificity index used to characterize per capita household consumption expenditure varies. Even when per capita household spending is similar, fulfillment propensity can differ across communities, depending on residents' awareness of the family doctor contract policy and their level of trust in physicians. Current management approaches rely on retrospective statistical data collection, resulting in significant information delays, hindering accurate forecasting of contract fulfillment, and limiting precise policy implementation. Clarifying the effect of per capita household consumption expenditure on the propensity to fulfill contracts is thus essential. Doing so can improve resource allocation, support the design of targeted incentive policies, capture regional macro-level trends in FDCS, and promote the sustainable development of primary healthcare systems.

Factor analysis (FA) is primarily used for dimensionality reduction and structural simplification. It explains the relationships among multiple observed variables by extracting latent variables (factors), identifying a small number of representative composite factors within a large set of variables, thereby reducing the number of variables. FA can serve as a multiparameter dimension reduction method for per capita household living expenditure, supporting analyses of household characteristics related to the fulfillment of FDCS (13). Backpropagation neural networks (BPNNs) employ adaptive learning to automatically extract patterns from data without predefined formulas. By adjusting weights through error backpropagation, they enable complex nonlinear modeling suitable for classification, regression, and prediction tasks. BPNNs demonstrate strong capabilities in nonlinear function approximation (14). Research integrating FA and BPNNs (FA–BPNNs) has been widely applied in healthcare studies. FA has served as a novel multiparameter selection method for per capita household living expenditure (15, 16), allowing analysis of the underlying relationships among eight aspects of household consumption. Findings indicate that established factor models significantly outperform conventional statistical methods in efficiency (17). Artificial intelligence theories and methods, represented by neural networks, have also been applied in research on family doctor services in primary healthcare (18) and on chronic disease management at the grassroots level (19), yielding favorable outcomes. Moreover, they have been used in analyses aimed at enhancing the quality of health care provided through FDCS (20). However, BPNNs employ gradient descent for optimization, rendering them prone to local optima and limiting their ability to converge to global optima (21). To address this limitation, the current study employed a GA–BP model to optimize initial connection weights and thresholds. Through FA of per capita household living expenditure, representative composite factors and their number were first extracted and then used as input for BP simulations of contract fulfillment across communities. This approach investigates the combined use of FA and BP algorithms for monitoring the fulfillment of FDCS. The aim is to provide insights and methodological guidance for community-level monitoring of contract fulfillment.

Research reveals that household consumption behavior constitutes a complex social behavioral system. Per capita household expenditure is far from a simple linear summation. Across the eight categories of personal consumption identified: food, tobacco, and alcohol; clothing; housing; household goods and services; transportation and communication; education, culture, and entertainment; healthcare; and other goods and services, these expenditures interact more like a sophisticated, dynamic ecosystem. Per capita consumption expenditure decisions are influenced by numerous intertwined factors, which exhibit complex interactions and multidimensional variable coupling. Healthcare expenditures constitute a rigid component of household consumption, influenced by both household health status and consumption capacity. The adoption of family doctor contract services is underpinned by community healthcare systems while also constrained by household spending capacity. It is precisely this nonlinear, multidimensional coupling that renders traditional linear regression models inadequate for accurately capturing underlying patterns, necessitating more robust analytical methods. To analyze these issues, we further examined relevant literature. In primary healthcare compliance or predictive modeling studies, we have compiled a literature review as shown in Table 1.

**Table 1:** Literature of methods on primary care fulfillment or predictive modeling.

**Literature and author**	**Proposed method**	**Research focus**
Saw et al. (25)	Structural equation modeling	Confirmatory factor analysis
Roi-Teeuw et al. (26)	Logistic regression	Prognostic factor study
Kitsellart et al. (27)	Data-driven and theory-driven	Statistics and research methods
Malo et al. (28)	Cluster analyses	Risk factors
Barrot et al. (29)	GLM, GLMs-Lasso, GBM, and SVM	Prognostic factors, predictors
Wang et al. (30)	Random forest model machine learning	Predicting
Pan et al. (31)	XGBoost, DT, RF, Light GBM, BP	Construct and validate models
Barsasella et al. (32)	Neural Network BP Algorithm	Predicting

This study analyzes the effect of per capita household living expenditure on the fulfillment of family doctor contracts. It explores the underlying mechanism of this influence by constructing and analyzing a dynamic model relationship between household expenditure and contract fulfillment across different communities. By identifying general patterns, this study provides decision-making support for the management of primary community health services. The contributions of this study are as follows:

(1) Factor analysis was employed to simplify the relationships embedded within per capita household expenditure data, achieving data reduction and noise removal. This clarified the complex interrelationships among household expenditure factors and highlighted key determinants.
(2) Based on a data-driven model, the learning advantages and generalization capabilities of artificial neural networks were utilized to conduct predictive analysis of the quantitative relationship between per capita household expenditures and family doctor contract fulfillment propensity.
(3) To overcome the sensitivity of the neural network's BP algorithm to initial weights and thresholds, which can lead to local optima, model optimization was performed using a genetic algorithm.
(4) It is pointed out that the key factor that constitutes the tendency of residents' families to fulfill the contract for family doctor services is the variable of benchmark living security or irreplaceable consumption, and it proposes recommendations to enhance the fulfillment rate of FDCS.

## 2 Materials and methods

### 2.1 Experimental design

**Figure 1:**
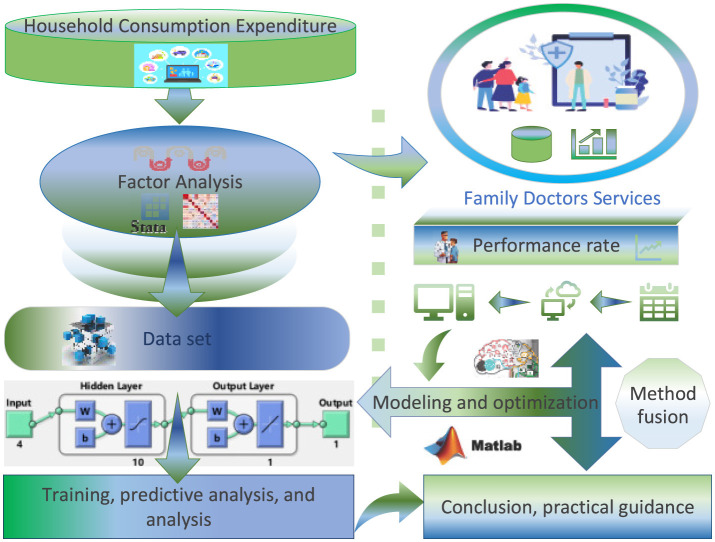
Experimental procedure.

This study was conducted between 2024 and 2025 in the Anning District of Lanzhou, Gansu Province. A total of 39 representative communities were selected, including the following: university communities where faculty and staff constitute the majority of residents, enterprise communities dominated by factory workers, residential communities formed by urban development-displaced populations transitioning into new urban residents, and large commercial district communities primarily inhabited by migrant populations. Data collected comprised eight major categories of per capita consumption expenditure across all communities in 2023: (i) food, tobacco, and alcohol; (ii) clothing; (iii) housing; (iv) household goods and services; (v) transportation and communication; (vi) education, culture, and entertainment; (vii) healthcare; and (viii) other goods and services. In addition, fulfillment data for FDCS were obtained from community health service centers, covering all quarters of 2024 and the first two quarters of 2025. FA was employed to conduct using Stata software to simplify variables representing per capita household consumption expenditure and the size of the service population across communities. Predictive models were established based on fulfillment rates for each quarter of 2024 and Q1–Q2 2025. The primary experimental workflow is illustrated in Figure 1. The experimental platform used Stata Statistics and Data Science **Stata/MP 18.0 for Windows Parallel Edition** and **MATLAB R2021b**, running in the following hardware and software environment: Lenovo PC with an 11th Gen Intel Core i7 2.80GHz and Microsoft Windows 11 Pro operating system.

### 2.2 Acquisition of per capita household consumption expenditure data

Per capita household consumption expenditure data for the 39 selected communities were obtained from the relevant statistical departments (Table 2).

**Table 2:** Composition of per capita household living expenditures.

**Consumer expenditure parameters**	**Variable name**	**Data type**
Food, tobacco, and alcohol	Food	Float
Clothing	Clot	Float
Housing	Habi	Float
Household goods and services	Home	Float
Transportation and communication	Tran	Float
Education, culture, and entertainment	Cult	Float
Healthcare	Heal	Float
Other goods and services	Othe	Float
Population served	Ser_pop	Int

These data are summarized in Table 3.

**Table 3:** Per capita household consumption expenditure in 39 communities.

**Community**	**Ser_pop**	**Food**	**Clot**	**Habi**	**Home**	**Tran**	**Cult**	**Heal**	**Othe**
C01	25768	10265.38	3092.23	8105.06	2177.36	9278.61	5634.05	5023.34	1298.07
C02	10,969	6583.49	1724.44	5345.31	811.29	4768.62	2020.66	2443.13	746.9
C03	8,001	4937.17	1118.27	4974.36	899.05	3792.27	1575.38	1500.56	666.75
C04	6,578	4975.49	1571.17	3111.29	773.65	3203.03	2402.04	1363.23	741.2
C05	11,813	6161.36	1375.55	3754.5	753.04	4476.26	2743.79	2245.81	874.55
C06	11,400	6421.9	1693.49	4064.45	909.42	4311.67	2349.01	2114.55	1255.09
C07	9,679	5973.06	1372.9	3161.43	774.22	4021.94	2200.02	2484.46	1020.91
C08	10,762	5185.64	1529.79	5473.72	672.06	3995.96	2022.28	2174.23	812.87
C09	29,180	15689.09	2863.79	16612.44	2889.49	10532.29	5552.15	4556.56	2892.8
C10	12,338	9476.95	1513.41	5962.53	1462.73	6483.18	4160.34	2105.29	1561.28
C11	29,160	11699.38	2441.07	11869.94	2168.72	9331.76	4860.74	3610.08	1652.46
C12	10,090	5740.28	1001.61	3797.9	923.24	3078.37	1833.53	1412.63	615.4
C13	10,437	9002.54	1418.98	5232.36	1179.16	5546.76	2306.79	1389.33	1242.89
C14	8,087	7181.65	889.59	3758.54	778	3303.15	1636.76	1338.18	709.49
C15	11,135	6590.47	1350.14	5403.3	1254.57	5033.8	2751.18	1841.9	836.1
C16	10,395	4897.27	1142.54	4876.46	872.93	3211.5	1375.04	1381.91	723.2
C17	10,090	7119.17	1015.65	3445.01	1064.2	3350.46	1839.75	1426.43	1128.24
C18	12,037	8063.17	974.39	4026.59	976.82	3322.57	1902.95	1755.01	1009.18
C19	25,262	10048.3	977.64	6043.96	1048.83	5282.65	1650.74	1590.32	1487.52
C20	10,307	6636.58	523.36	4389.46	718.47	2931.54	1116.62	1188.97	557.33
C21	13,315	6884.61	519.51	2423.1	596.97	2956.68	1450.28	762.41	850.62
C22	7,876	6623.2	821.12	2089.06	885.54	2487.22	1268.91	1345.04	453.71
C23	10,034	6909.69	943.44	2902.73	913.07	2878.14	1147.31	1394.35	610.53
C24	4,957	4805.62	598.88	2611.15	454.04	1841.61	953.84	632.83	396.8
C25	8,007	5904.51	677.66	4642.38	685.89	2582.33	1176.71	1339.86	446.4
C26	8,000	5197.62	1475.53	3317.64	853.02	1866.04	423.4	398.96	798.47
C27	16,832	4533.28	970.12	4058.83	683.65	3036.06	1971.91	1775.44	647.12
C28	7,373	4544.5	675.73	2338.58	585.07	2218.82	1352.63	1195.43	341.04
C29	15,000	5145.69	1155.13	2849.58	782.03	3481.32	874.97	1829.46	548.61
C30	12,568	4906.51	1109.72	3568.9	699.46	3167.4	1243.21	1910.2	791.85
C31	9,851	4519.9	1314	3525.1	585.39	2797.22	1076.6	1681.12	525.61
C32	26,548	13778.51	876.04	9887.57	1256.62	9013.9	679.04	1302.52	980.84
C33	28,373	9101.46	592.98	24416.11	1333.75	2476.5	1899	1014.69	558.49
C34	18,765	8362.32	1045.64	15312.34	945.26	3642.58	2426.06	1790	473.92
C35	20,151	10227.74	982.82	9052.68	1248.42	4561.12	3456.89	1397.81	502.27
C36	16,304	13899.04	1071.6	6241.68	1940.33	5294.21	2202.16	1964.75	485.42
C37	28,197	8593.41	848.88	18898.11	1538.27	2190.22	1819.22	1349.54	711.47
C38	13,084	6511.48	895.32	11114.73	960.37	1859.49	1491.78	2053.62	416.19
C39	20,120	14331.14	904.65	7893.73	564.52	4624.52	2441.54	1322.33	655.94

**Figure 2:**
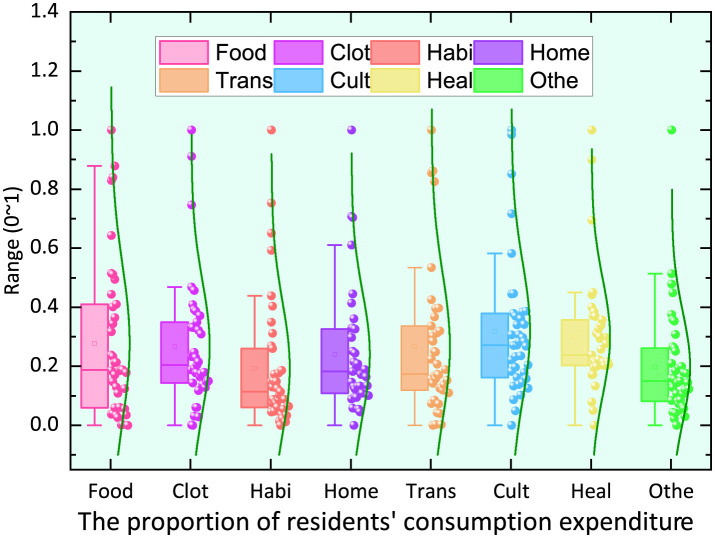
Box plot.

Box-and-whisker plots for each component are shown in Figure 2.

### 2.3 Performance data survey

After per capita household consumption expenditure data were collected, a concurrent survey was conducted to assess the fulfillment of family doctor contracts among community residents. Statistics were drawn from community health centers. The fulfillment rate was calculated as the percentage of community fulfillment visits relative to the total community service population. On the basis of the expected fulfillment targets for FDCS management, fulfillment rates were categorized into four levels—excellent (≥75%), good (≥50%–75%), fair (≥25%–50%), and poor (< 25%), which correspond to grades A, B, C, and D, respectively. Fulfillment rates per household were calculated using Equation (1).


$$\begin{array}{l} p_{-}r\%\text{=}\frac{\overset{6}{\underset{i=1}{\sum }}Q_{i}}{Ser_{\text{\_}}pop}\text{/1.5}\times \text{2.89}\times \text{100\%}, \end{array}$$ (1)

where *p*_−_*r%* represents the annual household fulfillment rate, 1.5 denotes the period (in years) corresponding to six quarters, and 2.89 is the average household population across the city (Data source: *Lanzhou Bureau of Statistics, 2023 Statistical Bulletin on National Economic and Social Development*).

The detailed results are presented in Table 4. In the table, Q1, Q2, Q3, and Q4 denote the four quarters of the year, and 2024 and 2025 indicate the respective years. The parameters correspond to the total number of contract fulfillment instances by community residents during each period.

**Table 4:** Per capita household consumption expenditure in 39 communities.

**Community**	**Ser_pop**	**Q1_24**	**Q2_24**	**Q3_24**	**Q4_24**	**Q1_25**	**Q2_25**	**Performance rate**	**Category**
C01	25,768	913	669	276	1,372	186	699	30.77%	C
C02	10,969	418	229	476	706	134	716	47.06%	C
C03	8,001	243	376	631	248	537	749	67.04%	B
C04	6,578	366	406	551	981	337	1,530	122.17%	A
C05	11,813	406	264	385	449	1,039	442	48.68%	C
C06	11,400	402	749	618	810	1,148	743	75.55%	A
C07	9,679	796	470	440	315	1506	1,172	93.54%	A
C08	10,762	791	621	354	290	8,87	159	55.53%	B
C09	29,180	828	315	584	365	927	1,080	27.06%	C
C10	12,338	423	199	627	519	516	1,894	65.24%	B
C11	29,160	313	186	563	790	620	726	21.13%	D
C12	10,090	800	230	568	1,175	1,342	1,031	98.26%	A
C13	10,437	786	393	377	627	132	800	57.50%	B
C14	8,087	351	699	442	1,726	526	238	94.87%	A
C15	11,135	820	525	607	571	153	547	55.77%	B
C16	10,395	641	390	254	1,890	793	1,303	97.70%	A
C17	10,090	302	587	743	945	898	1,310	91.37%	A
C18	12,037	311	591	679	1,112	104	791	57.43%	B
C19	25,262	575	429	631	1,782	238	689	33.13%	C
C20	10,307	571	555	690	963	585	618	74.43%	B
C21	13,315	373	522	363	1,035	289	816	49.17%	C
C22	7,876	260	597	249	858	1,159	1,281	107.73%	A
C23	10,034	849	883	395	585	1,189	1482	103.36%	A
C24	4,957	634	292	330	410	580	2,239	174.32%	A
C25	8,007	754	610	188	323	537	635	73.32%	B
C26	8,000	766	459	374	1,076	1,059	784	108.81%	A
C27	16,832	431	291	426	2088	223	618	46.67%	C
C28	7,373	765	213	105	233	1,827	875	105.00%	A
C29	15,000	338	386	451	1,227	1,875	1,019	68.02%	B
C30	12,568	331	349	255	555	451	946	44.26%	C
C31	9,851	486	287	616	1,007	359	685	67.28%	B
C32	26,548	258	673	138	962	1,043	329	24.70%	D
C33	28,373	412	146	504	917	378	738	21.02%	D
C34	18,765	631	922	549	1,597	371	231	44.16%	C
C35	20,151	546	531	236	582	847	614	32.09%	C
C36	16,304	661	815	232	544	958	586	44.86%	C
C37	28,197	300	182	355	618	537	2,382	29.89%	C
C38	13,084	839	422	918	1,104	1,564	1,238	89.60%	A
C39	20,120	255	904	465	1,976	934	2,683	69.11%	B

### 2.4 Data analysis and utilization

#### 2.4.1 Processing of raw data on per capita household consumption expenditure

Before FA was conducted, the data were tested to ensure they met the fundamental assumptions of the method: multivariate normality, correlation, and absence of multicollinearity. The Kaiser–Meyer–Olkin (KMO) test and Bartlett's test of sphericity were performed. The KMO statistic ranges from 0 to 1. A value close to 1 indicates strong correlations among variables, suggesting the suitability of the data for FA. Typically, a KMO value exceeding 0.6 is considered acceptable. In addition, a statistically significant result from Bartlett's test of sphericity (*p* < 0.05) confirms that the variables are sufficiently correlated to be suitable for FA. The specific values of the sampling adequacy tests are listed in Table 5.

**Table 5:** Kaiser–Meyer-Olkin measure of sampling adequacy.

**Variable**	**kmo**
Ser_pop	0.7912
Food	0.7987
Cloth	0.8369
Habi	0.6582
Home	0.9243
Trans	0.8375
Cult	0.9294
Heal	0.8634
Othe	0.9401
Overall	0.8506

The KMO values for all variables exceeded 0.6, with an overall KMO value of 0.8506, and the Bartlett's sphericity test was significant at the 0.05 level, indicating suitability for FA.

#### 2.4.2 Standardization of per capita household consumption expenditure data

For FA, the data were standardized using the system's default Z-score normalization method, as shown in Equation (2). This procedure transforms the data into a form with a mean of 0 and a variance of 1, eliminating the effect of varying measurement scales on the analysis.


$$\begin{array}{l} x^{\prime }=\frac{\left(x-\mu \right)}{\sigma } \end{array}$$ (2)

where μ denotes the mean, and σ represents the standard deviation.

For the Artificial Neural Network–Backpropagation (ANN–BP) algorithm, the data were normalized using min–max normalization, as shown in Equation (3). This technique linearly transforms the data into the interval [0, 1] for normalization.


$$\begin{array}{l} x^{\prime }=\frac{\left(x-x_{\min}\right)}{\left(x_{\max}-x_{\min}\right)}, \end{array}$$ (3)

where *x* is the raw data, and *x*_min_ and *x*_max_ represent the minimum and maximum values in the dataset, respectively.

#### 2.4.3 Factor analysis and GA-BP

The general FA model is given in Equation (4).


$$\{\begin{array}{l} x_{1}=\mu_{1}+a_{11}f_{1}+a_{12}f_{2}+\cdots +a_{1m}f_{m}+\varepsilon_{1}\\ x_{2}=\mu_{2}+a_{21}f_{1}+a_{22}f_{2}+\cdots +a_{2m}f_{m}+\varepsilon_{2}\\ \vdots \\ x_{p}=\mu_{p}+a_{p1}f_{1}+a_{p2}f_{2}+\cdots +a_{pm}f_{m}+\varepsilon_{p} \end{array},$$ (4)

where *f*_1_, *f*_2_, …, *f*_*m*_ and are common factors, while ε_*i*_ is the unique specific factor for variable *x*_*i*_(*i* = 1, 2, ⋯, *p*). *a*_*ij*_(*i* = 1, 2, ⋯, *p*; *j* = 1, 2, ⋯, *m*) represents the loading of variable *x*_*i*_ on common factor *f*_*i*_. The matrix form of the factor analysis model is shown in Equation (5).


$$\begin{array}{l} x=\mu +Af+\varepsilon , \end{array}$$ (5)

where *A* = *a*_*ij*_ is referred to as the factor loading matrix, $f={\left(f_{1},f_{2},\cdots ,f_{m}\right)}^{\prime }$ is the common factor vector, and ε = (ε_1_, ε_2_, ⋯, ε_*p*_)' is the specific factor vector.

FA was conducted on average household consumption expenditure data from the surveyed communities using Stata. The critical number of factors was determined based on two criteria: (i) retaining factors where each eigenvalue exceeded 1 and (ii) the cumulative contribution rate surpassed 80%. The analysis then yielded the factor data for further modeling. The ANN–BP model was implemented using the Neural Network Toolbox, consisting of a three-layer architecture: input, hidden, and output layers. The input vector was defined as *I*_−*input*_ and the desired output target as *O*_−*output*_. The input layer (denoted as *l*) comprised the number of composite factors with high contribution rates obtained from FA. The output layer (denoted as *n*) consisted of neurons, and the hidden layer contained neurons determined as $m=\sqrt{l+n}+\alpha \left(\alpha =1\sim 10\right)$.

#### 2.4.4 Data utilization

FA was conducted on all per capita household consumption expenditure data to achieve dimensionality reduction. For the dataset comprising per capita household consumption expenditure and household doctor contract service fulfillment data, the first 30 samples were used for system training, and the remaining nine samples for system testing. Actual prediction outputs were compared with expected outputs and visualized using an error plot. To implement differentiated management and service policies based on the classification of community fulfillment levels, predictive analysis was conducted on the fulfillment status of each community. Using classification data as training samples, various community types were simulated for forecasting.

## 3 Results analysis

### 3.1 Factor analysis of parameter dimension reduction

**Figure 3:**
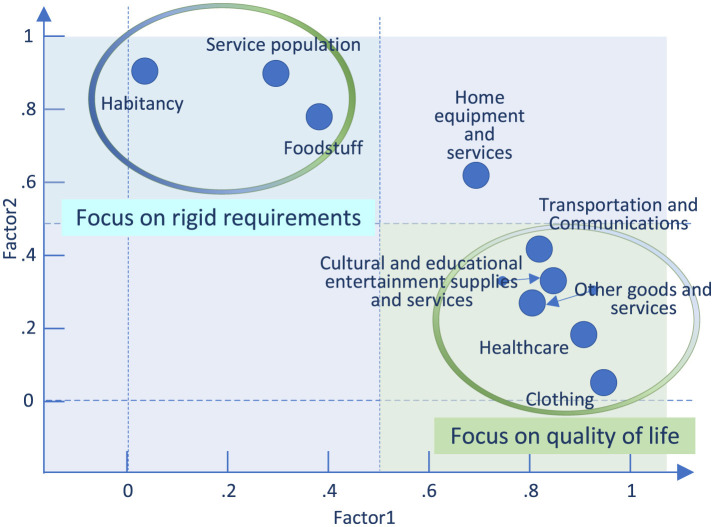
Factor loadings plot after factor rotation.

This study considered nine variables: community FDCS population (/ser_pop), food and tobacco (/food), clothing (/cloth), housing (/habi), household goods and services (/home), transportation and communication (/trans), education, culture, and entertainment (/cult), healthcare (heal), and other goods and services (/othe). A total of 39 samples were analyzed. Two factors were extracted and retained. LR testing of the model yielded a chi-square value of 347.51, with a *p*-value of 0.0000, indicating high statistical significance. In total, nine factors were extracted. The eigenvalues of the first two factors were 5.88282 and 1.52672, both exceeding 1, with a cumulative variance contribution of 82.33%. Factor 1 primarily explains /home /trans /cult /heal /othe /cloth /food /ser_pop, whereas Factor 2 mainly explains /habi /ser_pop. The variables not fully captured by the first two principal factors exhibited minimal information loss. After rotation of the factor structure, the variances of the two principal factors were 4.43331 and 2.97624, respectively. The factor loading plot after rotation is shown in Figure 3.

**Figure 4:**
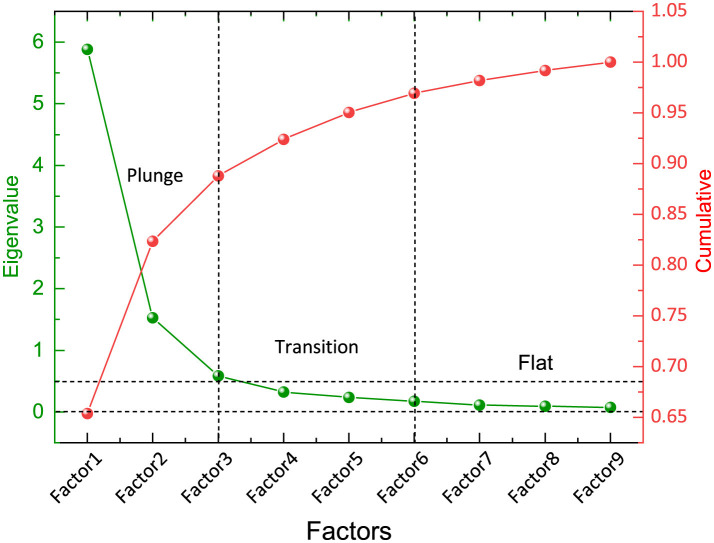
Scree plot.

As shown in Figure 4, the scatter plot shows the initial eigenvalues for each group of factors. The magnitude of each eigenvalue reflects the amount of information carried by the sample data in the new parameter coordinates. The first two factors exhibit eigenvalues greater than 1, indicating that they have substantial information. Subsequently, a transitional phase spans three factors, with eigenvalues approaching 0.5, followed by a gradual reduction in Factors 6–9. This trend is closely mirrored by the cumulative variance contribution curve for the extracted factors.

The factor score coefficient matrix is presented in Table 6.

**Table 6:** Factor score coefficient matrix.

**Variable**	**Factor1**	**Factor2**
ser_pop	–0.10732	0.37431
Food	–0.05218	0.29663
Cloth	0.301	–0.1902
Habi	–0.19603	0.43702
Home	0.0885	0.14538
Trans	0.17528	0.01891
Cult	0.20429	–0.03017
Heal	0.25726	–0.11613
Other	0.20405	–0.04956

### 3.2 Correlation analysis between per capita household consumption expenditure and contract performance

Drawing on the analysis in Section 2.1, this study introduces the concept of key contributing variables by selecting variables with significant contribution rates to the principal factors. Principal Factor 1 includes variables related to clothing (/cloth), culture (/cult), and health (/heal). These variables primarily reflect a community's happiness index or quality of life. Principal Factor 2 consists of variables related to services for the populace (/ser_pop) and housing (/habi), largely reflecting baseline living security or non-substitutable consumption. The analysis of the influencing factors reveals that the two principal factors, summarized as the “Quality of Life Factor” and the “Rigid Demand Factor,” accurately reflect real-world realities. The relationship between fulfillment rate and per capita household consumption expenditure was constructed using five variables. These variables were selected from an original nine-dimensional dataset and include the following: community service population (ser_pop); clothing (/cloth); housing (/habi); education, culture, and entertainment (/cult); and healthcare (/heal).

### 3.3 GA-BP simulation and testing of per capita consumption expenditure and compliance rate

**Figure 5:**
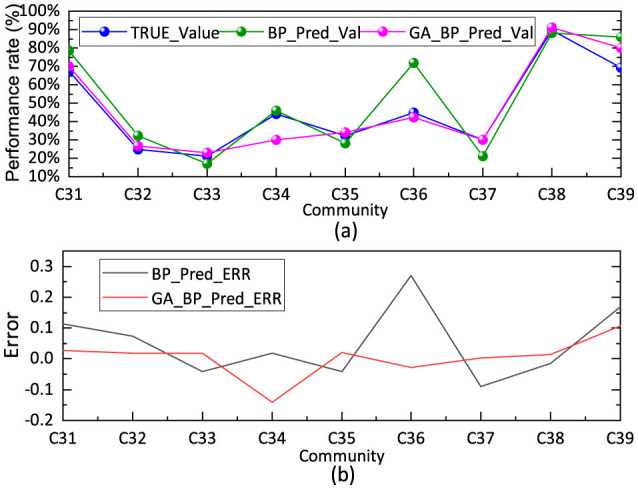
Results of GA-BP simulation prediction. **(a)** Compares the desired output with the actual output. **(b)** Presents the error output.

The input vector for the neural network was defined as the per capita household consumption expenditure variables extracted in Section 3.2 after FA, yielding an input dimension of 5. The objective function was the FDCS fulfillment rate among community residents, as described in Section 2.3. The number of hidden layer nodes was set to 9, corresponding to the number of public factors identified in the expenditure data. Thus, the constructed neural network model has a “5-9-1” architecture. The weights and activation function thresholds of the network were set to default values, and the learning process was iterated 1,000 times. After neural network training, the simulated predicted values were compared against the actual values. Figure 5 presents the GA–BP simulation prediction results for per capita household consumption expenditure and fulfillment rate.

Figure 5a compares the desired output with the actual output, alongside results predicted by the unoptimized BP algorithm. Figure 5b presents the error output. The results indicate that the coefficient of determination $R_{GA\text{\_}BP}^{\text{2}}=0.9223$ for the GA–BP fitted values relative to the measured values reached 1.0. The root mean square error (RMSE) and relative error (RE) of the fitted model were 0.0618 and 9.20%, respectively. This performance is significantly stronger than that obtained using the BP algorithm, achieving coefficients of determination of $R_{BP}^{\text{2}}=0.\text{7028}$, *RMSE* = 0.1207, and *RE* = 22.04%. The FA–GA–BP prediction model for the propensity to fulfill FDCS among residents thus demonstrates superior accuracy, relative to the conventional techniques that integrate parameter simplification with fulfillment rates but exclude FA. Moreover, the computation time decreased by 0.1032 s, indicating improved efficiency.

### 3.4 FA-GA-BP simulation and verification of per capita expenditure and compliance rates in different classification communities

**Figure 6:**
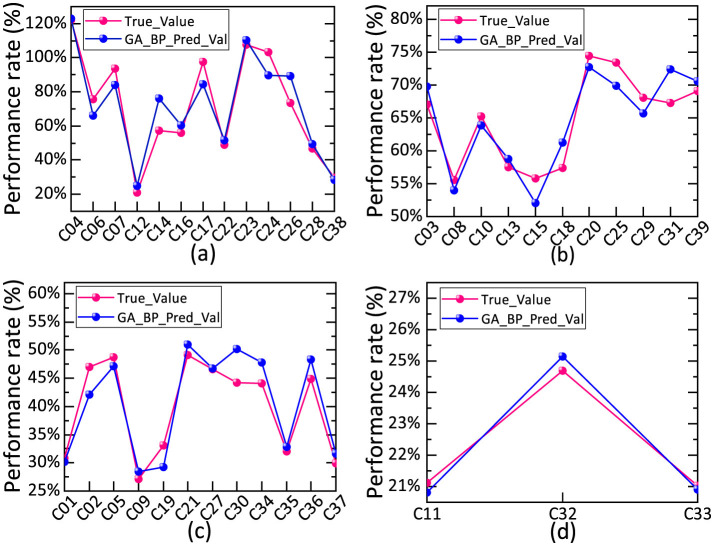
Simulated projections of per capita consumption expenditure and fulfillment rates across different classification communities. **(a)** Compares the simulated predictions with actual results for Class A communities. **(b)** Compares the simulated predictions with actual results for Class B communities. **(c)** Compares the simulated predictions with actual results for Class C communities. **(d)** Compares the simulated predictions with actual results for Class D communities.

The fulfillment rates were categorized as described in Section 2.3 to facilitate the adjustments and optimization of management measures across different fulfillment levels. After the classification results in Table 3 were obtained, the entire dataset was used for modeling. The fulfillment status corresponding to the per capita household consumption expenditure for each category served as the test data for predictive analysis. Following the analysis in Section 3.2, the FA–GA–BP model was structured as follows: input data, five dimensions (primary indicators of per capita household consumption expenditure after FA reduction); output data, six dimensions (fulfillment frequency for Q4 2024 and Q1–Q2 2025); model structure, “5-9-6.” The fulfillment rates for community households were calculated using Equation (1) and then compared based on the classifications in Table 3 of Section 1.3. Statistical analysis of the results followed. Figure 6 presents the FA–GA–BP simulation results for per capita consumption expenditure and fulfillment rates across different community classifications. Figure 6a compares the simulated predictions with actual results for Class A communities, and Figures 6b, c, d correspond to Class B, Class C, and Class D communities, respectively.

**Figure 7:**
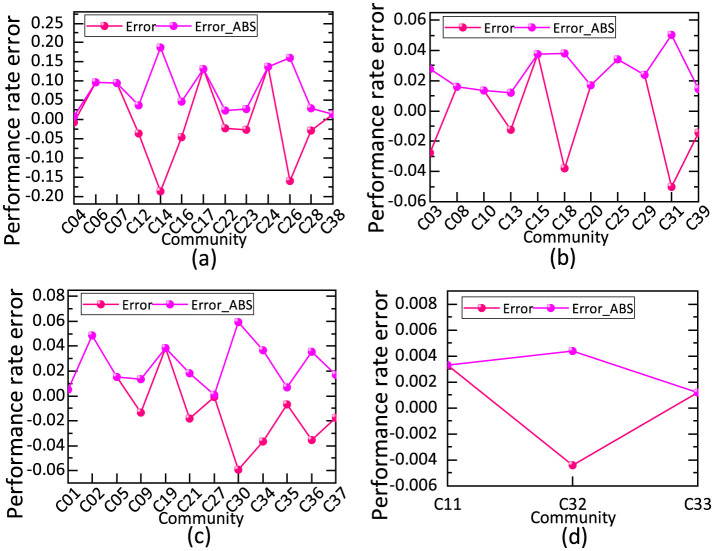
Simulation prediction error of per capita consumption expenditure and fulfillment rates across different classification communities. **(a)** The prediction errors of the proposed method for Class A community. **(b)** The prediction errors of the proposed method for Class B community. **(c)** The prediction errors of the proposed method for Class C community. **(d)** The prediction errors of the proposed method for Class D community.

The prediction errors of the proposed method were analyzed (Figure 7) for the four community categories A, B, C, and D.

**Figure 8:**
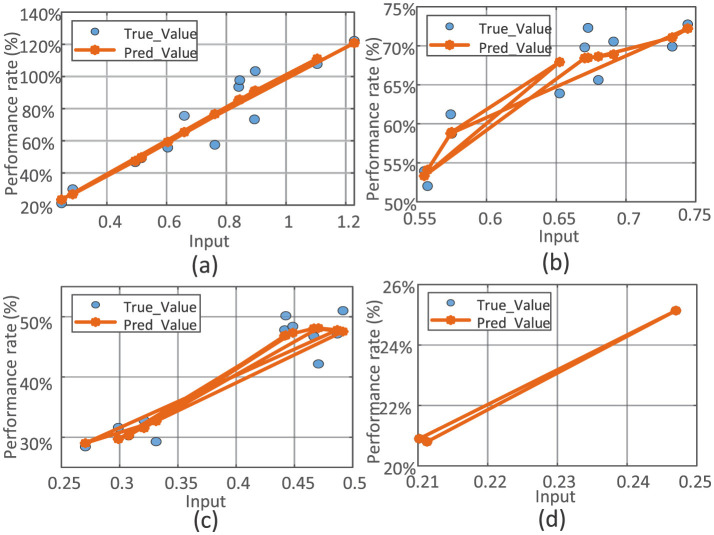
Scatterplot simulating per capita consumption expenditure and fulfillment rates across different classification communities. **(a)** Simulation result for category A. **(b)** Simulation result for category B. **(c)** Simulation result for category C. **(d)** Simulation result for category D.

Analysis of the simulation results for categories A, B, C, and D yielded scatter plots generated by polynomial nonlinear curve fitting, as shown in Figure 8.

The fit indices for each category were as follows: $R_{A}^{2}=\text{0}\text{.8987}$, *RMSE*_*A*_ = 0.0964, and *RE*_*A*_ = 11.39% for Class A; $R_{B}^{2}=\text{0}\text{.8138}$, *RMSE*_*B*_ = 0.0285, and *RE*_*B*_ = 4.04% for Class B; $R_{C}^{2}=\text{0}\text{.8571}$, *RMSE*_*C*_ = 0.0304, and *RE*_*C*_ = 6.12% for Class C; and $R_{D}^{2}=\text{0}\text{.9639}$, *RMSE*_*D*_ = 0.0033, and *RE*_*D*_ = 1.30% for Class D. Analysis of these results shows that the FA–GA–BP models outperformed the unclassified simulation models in predicting fulfillment rates. The simulated coefficient of determination for all categories exceeded 0.8138. Notably, in the Category D simulation, RMSE and RE were 0.0033 and 1.30%, respectively. These findings confirm that the FA–GA–BP model demonstrates high reliability and universality in predicting propensity to fulfill FDCS among residents.

## 4 Discussion

The propensity to fulfill FDCS among residents and the quality of such fulfillment are influenced by multiple factors. Among these factors, per capita household consumption expenditure is a multilayered, dynamic process that involves the interaction among socioeconomic dimensions, such as economic capacity, health awareness, and service demand (22). Economic conditions determine residents' access to services, directly influencing their capacity to pay. For low-income households, the basic payment costs of family doctor services constitute a substantial barrier. Data analysis indicates that when per capita consumption expenditure falls below 30% of the local average, the rate of contract renewal markedly declines. However, in studies examining the relationship between family doctor contract services and socioeconomic status, numerous scholars have engaged in relevant discussions and analyses. Researchers, including Huang Jiaoling (23), conducted a survey in a Shanghai community, finding that individuals with relatively lower socioeconomic status were more inclined to sign up for family doctor services. Analysing this through social stratification theory, they concluded that the cost-effectiveness of family doctors made them more readily accepted by lower socioeconomic groups. This aligns closely with the fundamental role of family doctors in providing primary healthcare services. The World Health Organization identifies primary healthcare as the most cost-effective healthcare model, widely adopted by numerous nations. Regarding these findings, the implementation of family doctor schemes varies considerably across regions due to differing economic conditions. However, Shanghai—as both a major economic hub in China and an early adopter of family doctor policies—continues to show a significant influence of socioeconomic status on family doctor enrollment. This underscores the enduring relevance and significance of this research. FA serves as a novel method for accurately identifying the effect of specific per capita consumption expenditures on family doctor contract fulfillment. This approach refines sensitive consumption expenditures to isolate the specific characteristics influencing fulfillment propensity. The integration of this approach with the advantages of the ANN–BP algorithm facilitates the precise prediction of fulfillment propensity. After a preliminary identification of sensitive sub-items in per capita consumer expenditure, this study conducts FA on these selected sensitive items to further refine the factors influencing fulfillment propensity: food, tobacco, and alcohol (/food); clothing (cloth); housing (/habi); household goods and services (/home); transportation and communication (/trans); education, culture, and entertainment (/cult); healthcare (/heal); and other goods and services (/othe). Among these factors, four were identified as core factors influencing a household's propensity to fulfill their FDCS contract: cult and heal for happiness indices or quality-of-life scales, and ser_pop and habi for basic living security or non-substitutable consumption. Together, these factors directly affect contract fulfillment. This finding aligns well with broader socioeconomic trends, including the public's pursuit of a higher quality of life and the practical requirements for basic social services to provide a safety net.

However, further research indicates that utilization levels of family doctor contract services vary among residents with different characteristics (24). Factors such as age, gender, household registration status, educational attainment, economic circumstances, and accessibility to healthcare services significantly influence residents' uptake of contracted services. This finding resonates with the present study's focus on socioeconomic status, collectively revealing that socioeconomic status exerts a substantial influence on willingness to fulfill obligations under the family doctor contract. Aggregate analysis may overlook specific nuances within certain segments. Categorizing FDCS fulfillment rates across different communities and constructing a fulfillment rate propensity analysis model can more fully reflect the diverse fulfillment rate propensities across communities. This approach reduces the noise from multifaceted factors such as per capita disposable income, yielding significantly superior equation-fitting results compared to aggregate-level analysis. It enables the design of targeted FDCS strategies tailored to the specific management needs of different communities.

This study employed the ANN–BP algorithm to simulate the fulfillment rate of FDCS. Testing of the model revealed a coefficient of determination equal to 0.9223 between the measured and predicted values. Through classification comparisons, the FA–GA-BP method effectively reverse-engineered the relationship between the per capita household expenditure and contract fulfillment propensity across different communities. The model demonstrated high determination coefficients, low error rates, and strong applicability across diverse classifications, making it crucial for assessing fulfillment status. Neural network simulations also achieved excellent fitting results in regional classification studies of family doctor contract fulfillment, with correlation coefficients exceeding 0.8138. However, BP algorithm training is susceptible to converging on local minima due to the BP neural network's sensitivity to initial weight settings and sample size. The GA algorithm employed in this study optimized the initial weights and thresholds of the BP neural network, yielding favorable results. Therefore, the findings demonstrate the potential advantages of integrating FA with the ANN–BP algorithm. The model inversion achieved strong overall performance, and GA optimization significantly enhanced precision. Accurate prediction of fulfillment tendencies can help explain how per capita household consumption expenditure shapes service participation across different communities. This approach has broad application prospects for rapidly monitoring and evaluating fulfillment tendencies, as well as for precisely implementing FDCS strategies within a region. Future monitoring efforts of FDCS fulfillment should focus on strengthening the stratified management of patient profiles and customizing personalized service packages for these contracts. By fully leveraging the ultra-early predictive capabilities of the per capita household consumption expenditure index, fulfillment rates can be significantly enhanced. With the development of intelligent support systems and continuous advancements in data analysis techniques, a more in-depth analysis of the economic and physical factors influencing the fulfillment and development of FDCS is needed. Such research would enable early detection and tracking, ultimately supporting the fulfillment goals of FDCS. Thus, this area warrants further study.

## 5 Limitations of the study

The statistics on contract fulfillment by community residents were collected quarterly. This segmentation ignores the effects of seasonal epidemics or other special circumstances that may influence fulfillment patterns. For instance, influenza outbreaks or variations in community demographics, such as a higher proportion of older adult residents or children, could significantly influence fulfillment rates.

## 6 Conclusions

This study examines the predictive effect of per capita household consumption expenditure on the fulfillment tendency of family doctor contract services, yielding the following primary conclusions:

(1) Compared with the more regular patterns of change in per capita household consumption expenditure, the variations in FDCS fulfillment tendency are less predictable. However, by simplifying the correlations among parameters via FA, the network's predictive performance improved significantly. FA also reduced input dimensionality, resulting in a more streamlined model.
(2) The integration of FA with the ANN–BP algorithm effectively simulated changes in contract fulfillment. The GA algorithm further optimized the BP neural network by addressing the issue of local optima arising from random weight and threshold initialization.
(3) The selection of principal components should not rely solely on eigenvalues. In this study, two principal components with eigenvalues greater than one were identified, yet their overall predictive performance was inferior to that of the initial parameters. Determining the number of principal components based on eigenvalue magnitude is equivalent to thresholding by a contribution rate. From the scree plot, principal component selection should also consider eigenvalue magnitude and descending trends. Based on the dimension reduction results for per capita household consumption expenditure parameters, this study suggests retaining eigenvalues from transitional segments and analyzing whether to retain or exclude smooth segment eigenvalues.
(4) Future efforts should establish a refined “monitoring–analysis–intervention” closed-loop system tailored to different communities. By aligning resident needs with policy guidance, this approach aims to transition from “passive enrollment” to “active fulfillment,” ultimately enhancing the precision and accessibility of primary healthcare services.

## Data Availability

The original contributions presented in the study are included in the article/supplementary material, further inquiries can be directed to the corresponding author.
